# MRI signal intensity per vertebral volume: a novel biomarker in pediatric osteoporosis

**DOI:** 10.1007/s11604-026-01967-x

**Published:** 2026-03-14

**Authors:** Ahmet Faruk Gürbüz, Ayşe Keven, İsmail Özgül, Sadi Elasan, Mesut Parlak, Can Çevikol

**Affiliations:** 1 Department of Radiology, Akdeniz University School of Medicine, Dumlupınar bulvarı, Arapsuyu, 07059 Antalya, Turkey; 2 Department of Biostatistics, Medical Faculty, Yüzüncü Yıl University, Van, Turkey; 3 Department of Pediatric Endocrinology, Akdeniz University School of Medicine, Antalya, Turkey

**Keywords:** Osteoporosis, Bone mineral density, Magnetic resonance imaging, Pediatric radiology, Metabolic bone diseases

## Abstract

**Purpose:**

This study aimed to evaluate the diagnostic performance of a novel MRI-based parameter—MRI signal intensity per unit vertebral volume—in identifying low bone mineral density (BMD) in children and adolescents, alongside established MRI metrics including mean L1-L4 signal intensity and vertebral bone quality (VBQ) score.

**Materials & methods:**

The study included 106 osteoporotic patients (aged 5–18 years) who had not yet reached 18 years of age, and 46 age-matched controls. Subjects were grouped into childhood (5–11 years) and adolescence (> 12 years). Using 1.5 T MRI, mean L1-L4 signal intensity, VBQ score, and signal intensity per unit volume were calculated. MRI findings were compared using t-tests and ANOVA; diagnostic accuracy was assessed via ROC curve analysis.

**Results:**

In the childhood group, all MRI parameters significantly differed between low bone mineral density and control subjects (*p* < 0.05). In adolescents, only mean signal intensity and signal intensity per unit volume were significant. The new parameter demonstrated the highest diagnostic value, with AUCs of 0.792 and 0.836 in childhood and adolescence groups, respectively.

**Conclusions:**

MRI signal intensity per unit vertebral volume showed superior performance in detecting low BMD compared to existing MRI-based indices. This parameter offers a radiation-free, size-adjusted alternative to DXA, particularly valuable in pediatric patients requiring long-term monitoring.

## Introduction

Osteoporosis is a metabolic disease characterized by reduced bone mineral density (BMD), leading to an increased risk of fractures. It may develop primarily due to genetic factors or secondarily as a result of chronic disease or its treatment [1]. In recent years, pediatric osteoporosis has been reported more frequently, especially among children with chronic illnesses, due to longer life expectancy, increased exposure to treatments affecting bone health, and advances in diagnostic imaging. Early recognition of osteoporosis in children and adolescents is essential for initiating timely intervention and minimizing fracture risk.

Bone marrow undergoes age-specific histological and radiological changes starting from birth. During childhood, marrow fat content is lower compared to adults. Magnetic resonance spectroscopy (MRS) studies have shown that vertebral fat content increases from 24% in the 11–20 age group to 54% after 61 years of age [2].

Although dual-energy X-ray absorptiometry (DXA) remains the most commonly used method for diagnosing osteoporosis, its applicability in pediatrics is limited due to its sensitivity to body size and the use of adult-based reference values [3]. Peripheral quantitative computed tomography (pQCT) and lumbar spine MRI are other modalities under consideration [1]. However, pQCT also involves ionizing radiation. MRI, in contrast, offers several advantages: it is radiation-free, provides high soft-tissue contrast, allows volumetric assessment, and correlates well with DXA-derived BMD and T scores. Osteoporotic changes in bone marrow, such as trabecular loss and adipocyte infiltration, manifest as increased T1-weighted signal intensity [4].

The Vertebral Bone Quality (VBQ) score is a recently introduced, MRI-based quantitative measure that reflects trabecular bone composition by evaluating signal intensity ratios between vertebral bodies and cerebrospinal fluid on routine T1-weighted images. Unlike dual-energy X-ray absorptiometry (DXA), which is the current standard for assessing bone mineral density, the VBQ score can be obtained opportunistically without additional imaging, radiation exposure, or cost. This makes it particularly useful in patients who undergo spinal MRI for various clinical indications. Growing evidence suggests that the VBQ score is associated with bone fragility and can predict vertebral fracture risk, serving as a surrogate marker for osteoporosis [5, 6]. The vertebral bone quality (VBQ) score, calculated as the ratio of mean vertebral signal intensity to cerebrospinal fluid signal at the L3 level on T1-weighted images, has gained popularity in assessing bone quality [6]. Although limited, some pediatric studies suggest its association with low BMD [7, 8]. Notably, VBQ does not account for vertebral volume, while DXA is known to be confounded by body size [3].

Given these considerations, our study aimed to assess the diagnostic value of a novel MRI-based parameter—MRI signal intensity per unit vertebral volume—in detecting low BMD in pediatric patients. Absolute MRI signal intensity values can be influenced by vertebral size, coil loading, and patient-specific factors, which may limit their reproducibility across individuals. To address this limitation, we devised the parameter *“MRI signal intensity per unit vertebral volume.”* By normalising signal intensity to vertebral volume, this approach aims to reduce inter-patient variability and enable a more standardised and comparable evaluation of bone quality. We compared its performance with VBQ and mean L1-L4 signal intensity, hypothesizing that this volumetric normalization would reduce the confounding effects of body size and offer a clinically meaningful, radiation-free alternative in pediatric osteoporosis assessment.

## Materials and methods

### Study design

The protocol of this single-center retrospective observational study was approved by the University’s ethics committee (Approval Date: 27.03.2025, Approval No: 311). The study was conducted in accordance with the ethical principles outlined in the Declaration of Helsinki, as revised in Edinburgh in 2000. Informed consent was obtained from the legal guardians of all patients included in the study.

### Population and sample

The study population consisted of 128 children with low bone mineral density aged between 5 and under 18 years of age who underwent lumbar MRI and DXA between February 2019 and January 2025 and had a DXA Z score of -2 and below. Six patients with a history of spinal surgery, four with spinal infection, seven with primary tumors in the spinal region, and five with insufficient MRI quality were excluded from the study. In the end, the study sample consisted of 106 children with low bone mineral density, 62 males and 44 females, and the control group consisted of 46 children and adolescents age-matched with the patient group, 26 males and 20 females, who underwent lumbar MRI and had a DXA Z score above − 2 during the study period. MRI and DXA examinations were paired if performed within ± 3 months. In our cohort, the mean interval was 41.4 days (median: 31.5; range: 0–113), with 50% of patients undergoing both examinations within 10–71 days (IQR: 61).

**Fig. 1:**
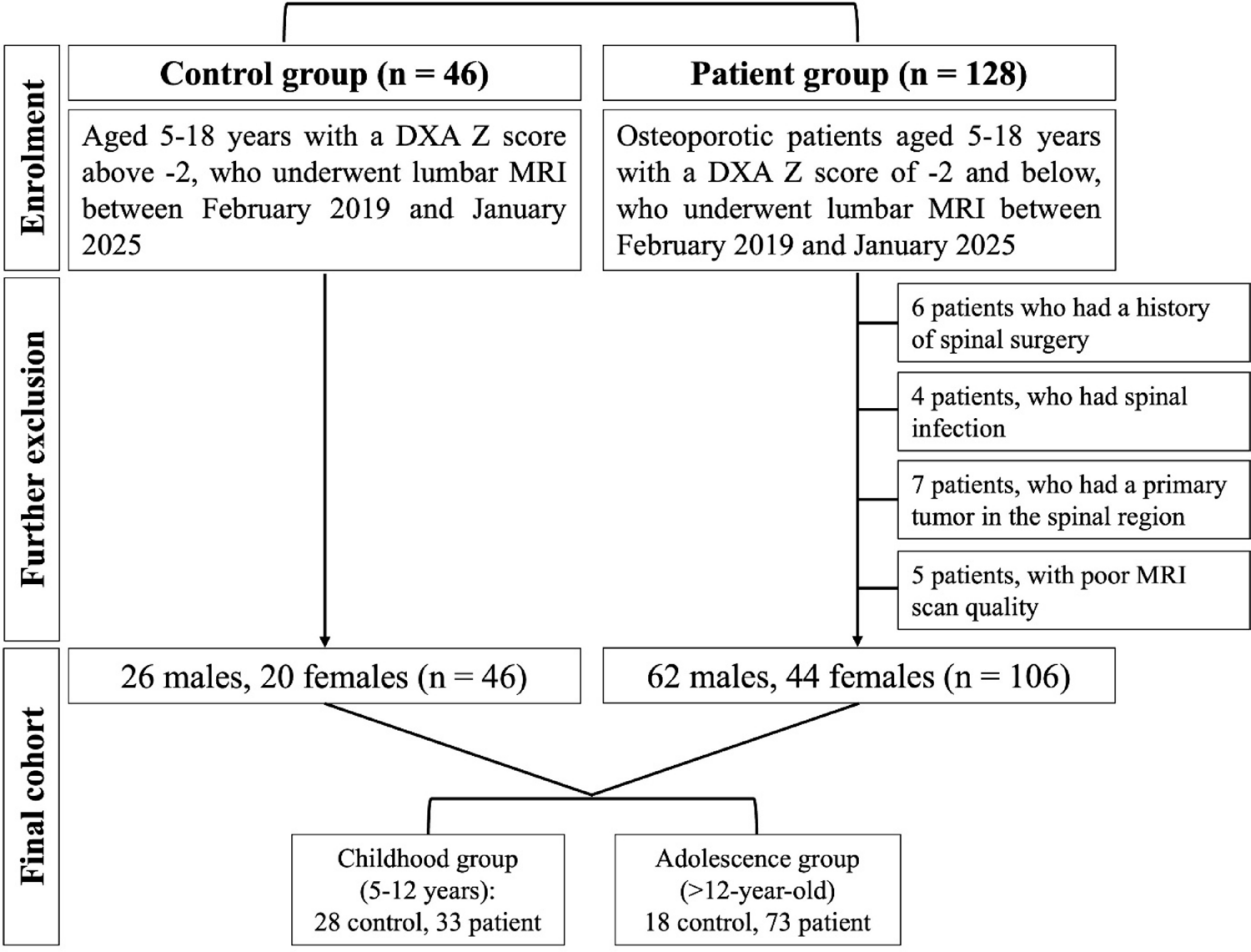
Flow diagram illustrating the systematic selection process and categorization methodology of patients. The final pediatric cohort was created by excluding cases with specific exclusion criteria from 128 potential cases identified in the initial screening

We used pediatric age ranges recommended by the United States Department of Health and the Food and Drug Administration (FDA) [9]. Accordingly, patient and control groups were further categorized into childhood (5–11 years old) and adolescence (> 12 years old) groups. There were 61 cases in the childhood group, 33 from the patient group and 28 from the control group, and 91 cases in the adolescence group, 73 from the patient group and 18 from the control group (Fig. 1).

Low bone mineral density patients’ age, gender, the primary disease for which they were being followed up, bone fracture history, solid organ or bone marrow transplantation history, corticosteroid use, body mass index (BMI), quantitative findings obtained from lumbar MRI by the radiologist and DXA parameters were recorded. Additionally, osteoporosis patients’ serum calcium (mg/dL), phosphorus (mg/dL), magnesium (mg/dL), parathormone (pg/mL), 25(OH)D vitamin (ng/mL), alanine aminotransferase (IU/L), alkaline phosphatase (IU/L), gamma-glutamyl transferase (IU/L), creatinine (mg/dL) and urine creatinine values were noted. On the other hand, control subjects’ age and gender and quantitative findings obtained by the radiologist from lumbar MRI and DXA parameters were recorded.

### MRI protocol and analysis

All participants underwent lumbar MRI in the supine position with a 1.5 T MRI device (Aera, Siemens, Erlangen, Germany) using a body coil.

The MRI sequence parameters applied were as follows: in sagittal T1-weighted imaging, repetition time (TR)/echo time (TE): 590/9, echo train length (ETL): 5, slice thickness: 3 mm, matrix: 300 × 320, field of view (FOV): 300 × 300 mm; in axial T2-weighted turbo spin echo sequence, TR/TE: 11,270/90, ETL: 17, section thickness: 4 mm, matrix: 320 × 320, FOV: 200 × 200 mm. Lumbar spinal images were evaluated collaboratively by two radiologists with 8 and 18 years of experience, respectively. Both readers reviewed the images together and reached a consensus on all measurements, which were then used for subsequent analyses. Consequently, interobserver variability was not assessed as measurements were obtained through joint agreement.

**Fig. 2:**
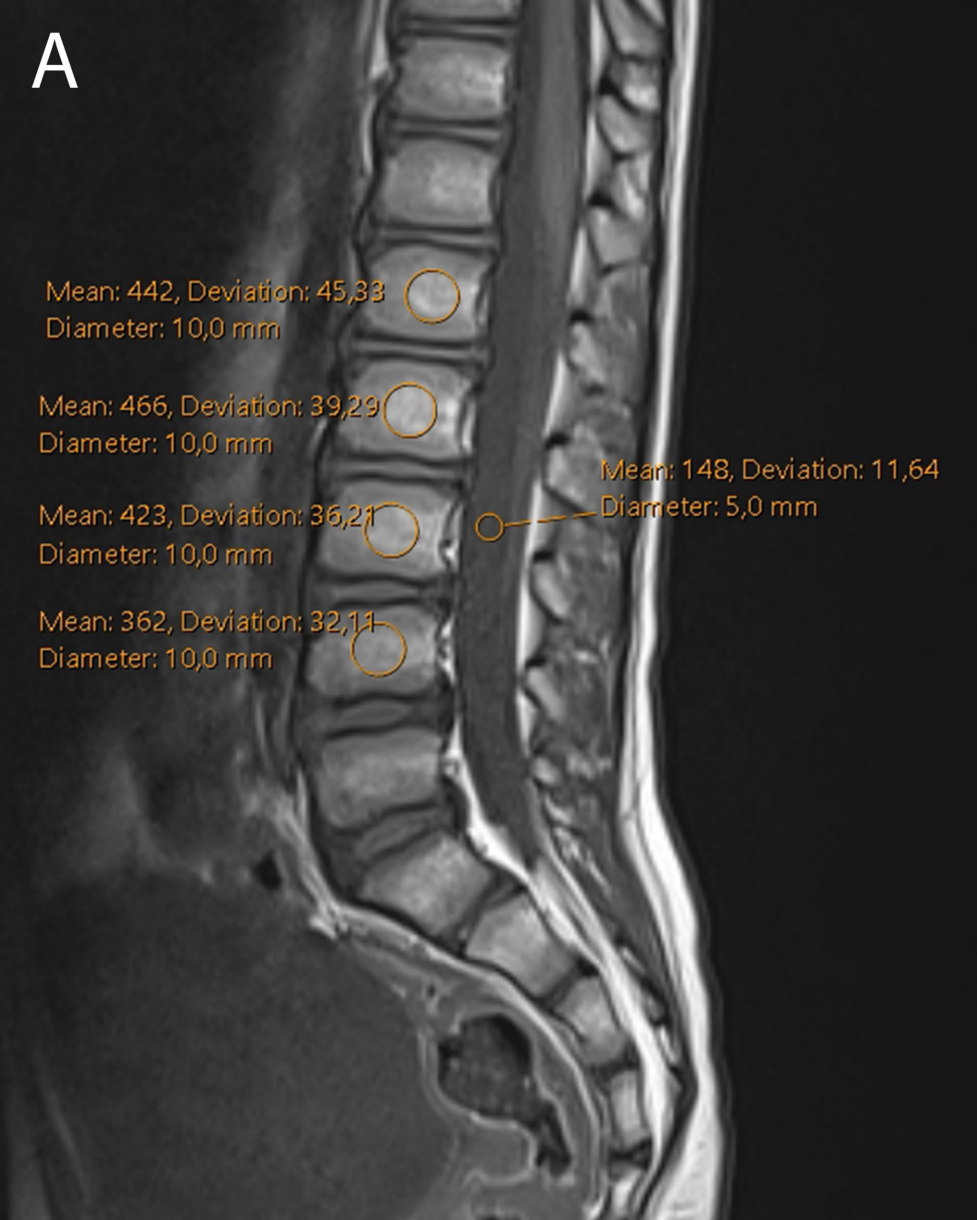

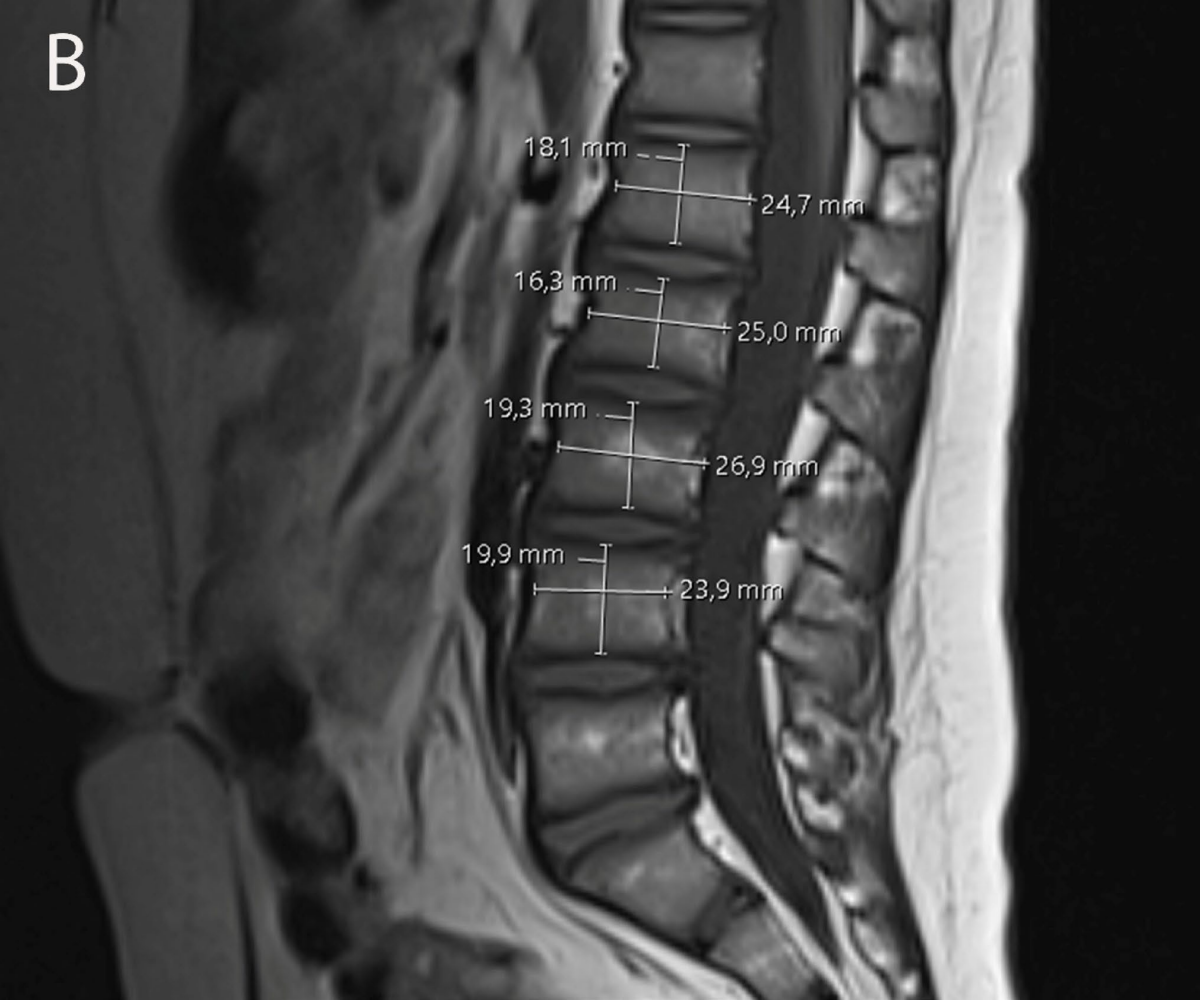

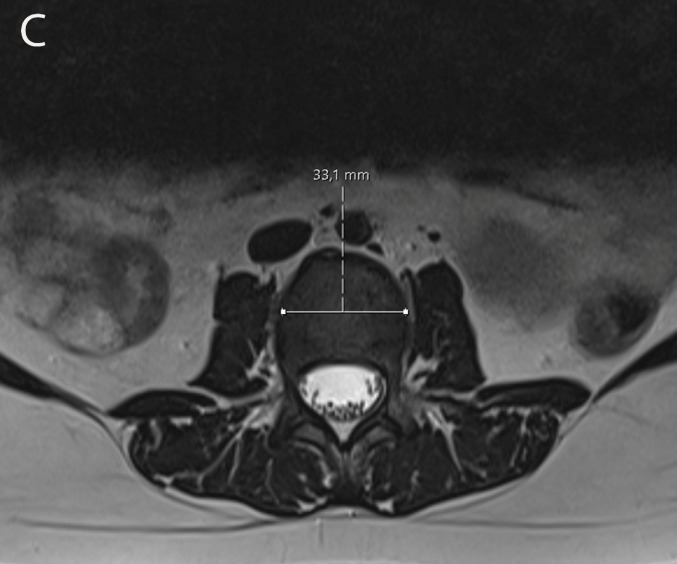
Vertebral MRI-based quantitative measurement methodology. **(A)** Signal intensities obtained by placing an ROI with a diameter of 10 mm in the L1-L4 vertebral bodies in T1-weighted midsagittal planes and reference measurements performed with cerebrospinal fluid at the L3 level. **(B)** Millimetric measurements of anteroposterior and longitudinal dimensions of vertebral bodies in the sagittal plane. **(C)** Measurements of transverse diameters passing through the equatorial plane of the vertebral body on axial T2-weighted sections. Volumetric calculations were carried out using these three orthogonal measurements

In T1-weighted sagittal plane imaging, the mean MRI signal intensities were recorded by placing a circular region of interest (ROI) with a diameter of 10 mm at the center of the L1, L2, L3, and L4 vertebral bodies in the midsagittal plane. The mean MRI signal intensities of the four vertebrae were summed and divided by four to calculate the meanT1-weighted signal intensity of the L1-L4 vertebra corpus (Fig. 2A).

The MRI signal intensity obtained by placing a circular ROI with a diameter of 5 mm into the cerebrospinal fluid (CSF) in the spinal canal at the level of the L3 vertebra was measured. The VBQ score was calculated by dividing the mean L1-L4 MRI signal intensity by the CSF signal intensity (Fig. 2A).

On sagittal T1-weighted and axial T2-weighted images, the transverse **(a)**, anteroposterior **(b)**, and longitudinal **(c)** dimensions of the L1-L4 vertebral bodies were measured in millimeters, and the volume of each vertebra (cm^3^) was calculated.

Vertebral volumes (VV) were calculated by multiplying the vertebral area in the axial plane by the vertebral height (VV: axbxc) [10, 11]. The calculated VVs were divided by four to obtain the mean L1-L4 VV. The MRI signal intensity per unit volume of the vertebral body was calculated by dividing the mean L1-L4 MRI signal intensity by the mean VV (Figs. 2A-C).

Thus, three MRI quantitative parameters were used in the study: mean L1-L4 vertebral body T1-weighted MRI signal intensity, VBQ score (L1-L4 average SI / CSF SI), and MRI signal intensity per unit volume of the vertebral body (mean L1-L4 signal intensity / mean L1-L4 volume).

### DXA protocol and analysis

Participants underwent lumbar spine (L1–L4) DXA examination in the supine position using a Norland XR-36 densitometer (Norland Instruments, Fort Atkinson, WI, US).

DXA examination of all cases was performed by the same certified technician using the same device. Z scores described by the International Society of Clinical Bone Density (ISCD) were used for the diagnosis of osteoporosis [12].

Participants’ age- and height-adjusted Z scores, i.e., height-for-age Z-scores (HAZ), were calculated using their age, gender, and DXA lumbar spine bone mineral density data ​​using the formula proposed by Zemel et al. [13]. Calculations were carried out on the open-access website at “https://zscore.research.chop.edu/calcpedbonedens.php”.

Based on these calculations, cases with HAZs below − 2, calculated using DXA lumbar spine bone mineral density, were included in the patient group, and cases with HAZs above − 2 and no vertebral fractures detected on MRI were included in the control group. DXA bone mineral contents, DXA bone mineral densities, and DXA Z scores for the cases in both groups were recorded. The mean Z scores of the patient and control groups were − 3.37 and − 0.69, respectively (*p* < 0.001).

### Statistical analysis

The sample size of the study was calculated using the G*Power statistical software (ver.3.1.9.7). It was determined that there should be a minimum of 46 cases in each of the patient and control groups in the independent t-test experimental design for 0.80 test power, 0.6 effect size (mean value in the literature) and 0.05 type-1 error (α). However, the number of cases was increased to ensure the test’s power. To this end, 106 cases were included in the patient group and 46 cases in the control group. Accordingly, the “post-hoc power value” was recalculated as 92%.

The conformity of continuous variables to normal distribution was assessed using Kolmogorov-Smirnov and Skewness-Kurtosis tests, and all parameters were found to be normally distributed. Independent t-test was used to compare MRI parameters between patient and control groups. One-way analysis of variance (ANOVA) was used to compare age groups (childhood and adolescence). Duncan’s test was preferred in post-hoc analyses.

ROC curve analysis was performed to evaluate the diagnostic performance of MRI parameters (mean L1-L4 MRI signal intensity value, VBQ score and MRI signal intensity per unit volume of the vertebral body ) in predicting low BMD, with area under the curve (AUC) values calculated. The Youden index was used to determine the optimal cut-off value maximizing sensitivity and specificity. Pearson correlation coefficients were used to determine relationships between radiological parameters and DXA Z-scores.

To account for the potential confounding effect of vertebral body volume on our primary outcome measure (MRI signal intensity per vertebral volume), we conducted an analysis of covariance (ANCOVA) in the Adolescent group, where greater variability in vertebral size was expected. In this model, SI/Volume was the dependent variable, group (Low BMD vs. Control) was the fixed factor, and age, sex, and vertebral body volume were entered as covariates. Adjusted group means and 95% confidence intervals (CIs) were derived from the model. Probability (*p*) statistics of < 0.05 were deemed to indicate statistical significance. Statistical analyses were performed using SPSS 26.0 (Statistical Product and Service Solutions for Windows, Version 26.0, IBM Corp., Armonk, NY, US, 2019) software package.

## Results

The most common primary disease in the patient group was acute lymphoblastic leukemia in remission (*n* = 19, 17.9%), followed by thalassemia major (*n* = 12, 11.3%), cystic fibrosis (*n* = 5, 4.7%) and congenital nephrotic syndrome (*n* = 4, 3.7%). In the patient group, 17 (16%) cases, 10 males and 7 females, had a history of bone fractures.

### Comparison of low bone mineral density patients with and without bone fracture history

There were no statistically significant differences between the demographic, radiological, and laboratory parameters of cases with and without a history of bone fracture in the patient group (Table 1). DXA Z scores and parathormone levels of patients with a bone fracture history were found to be lower, although not significantly, compared to patients without a bone fracture history (*p >* 0.101 for all parameters). In terms of laboratory parameters, it was found that 25(OH)D vitamin levels were below the reference range in both patient groups with and without a history of bone fracture, while other biochemical parameters were within normal limits in both groups.

**Table 1:** Distribution of Demographic, Radiological and Laboratory Characteristics of Osteoporosis Patients by the Patient Groups with and without Fracture History

	Fracture History		
Characteristics	Yes (*n* = 17)	No (*n* = 89)	*p* value
Demographic Characteristics			
Age *(year)*	13.88 ± 3.31	14.20 ± 3.25	0.711
*Gender*			
Male	10 (%16.1)	52 (%83.9)	0.976*
Female	7 (%15.9)	37 (%84.1)	
Body mass index *(kg/m*²)	18.71 ± 4.51	17.48 ± 3.46	0.206
*Radiological Characteristics* *MRI Parameters*			
Mean L1-L4 MRI signal intensity	267.69 ± 82.08	257.70 ± 90.37	0.673
VBQ score	2.54 ± 0.71	2.45 ± 0.73	0.66
MRI signal intensity per unit volume of the vertebral body	15.89 ± 7.04	17.09 ± 6.90	0.514
VBQ/Volume	22.69 ± 17.65	18.62 ± 12.18	0.245
*DXA Parameters*			
DXA BMC*(g)*	21.67 ± 8.72	23.03 ± 8.71	0.560
DXA BMD*(g/cm²)*	0.50 ± 0.14	0.53 ± 0.13	0.427
DXA T score	-5.67 ± 1.99	-5.34 ± 1.70	0.486
DXA Z score	-3.49 ± 0.80	-3.50 ± 1.80	0.983
*Laboratory Characteristics*			
Calcium *(mg/dL)*	9.60 ± 0.57	9.65 ± 0.57	0.767
Phosphorus *(mg/dL)*	4.39 ± 0.74	4.47 ± 0.68	0.678
Magnesium *(mg/dL)*	2.01 ± 0.22	2.06 ± 0.21	0.355
PTH *(pg/mL)*	34.06 ± 15.69	43.02 ± 20.50	0.101
25(OH)D *(ng/mL)*	28.59 ± 6.64	25.81 ± 11.08	0.320
ALT *(IU/L)*	18.94 ± 10.48	23.17 ± 20.07	0.400
ALP *(IU/L)*	202.12 ± 94.91	208.32 ± 90.02	0.802
GGT *(IU/L)*	19.94 ± 6.56	20.85 ± 10.50	0.733
Creatinine *(mg/dL)*	0.59 ± 0.32	0.76 ± 0.70	0.327
Urine creatinine	78.45 ± 48.06	86.81 ± 53.01	0.558

*Values ​​are given as mean ± standard deviation or n (%).Significance levels were calculated based on independent sample t-test results.*Calculated using the chi-square test.VBQ: Vertebral Bone Quality*,* DXA: Dual Energy X-ray Absorptiometry*,* BMC: Bone Mineral Content*,* BMD: Bone Mineral Density*,* PTH: Parathormone*,* ALT: Alanine Aminotransferase*,* ALP: Alkaline Phosphatase*,* GGT: Gamma-glutamyl Transferase*

### Comparison of low bone mineral density patients and control subjects in the childhood group

The childhood (5–11 years) group consisted of 61 cases, 33 of whom were from the patient group, 25 males and 8 females, and 28 from the control group, 11 males and 17 females. The mean DXA Z scores of children with low bone mineral density patients and control subjects were − 3.22 and − 0.77, respectively.

In terms of MRI parameters, mean L1-L4 MRI signal intensity (305.2 vs. 240.7; *p* = 0.002), VBQ score (2.7 vs. 2.3; *p* = 0.02), and MRI signal intensity per unit volume of the vertebral body (31.1 vs. 18.01; *p* < 0.001) were found to be significantly higher in the childhood patient group than in the childhood control group.

**Fig. 3:**
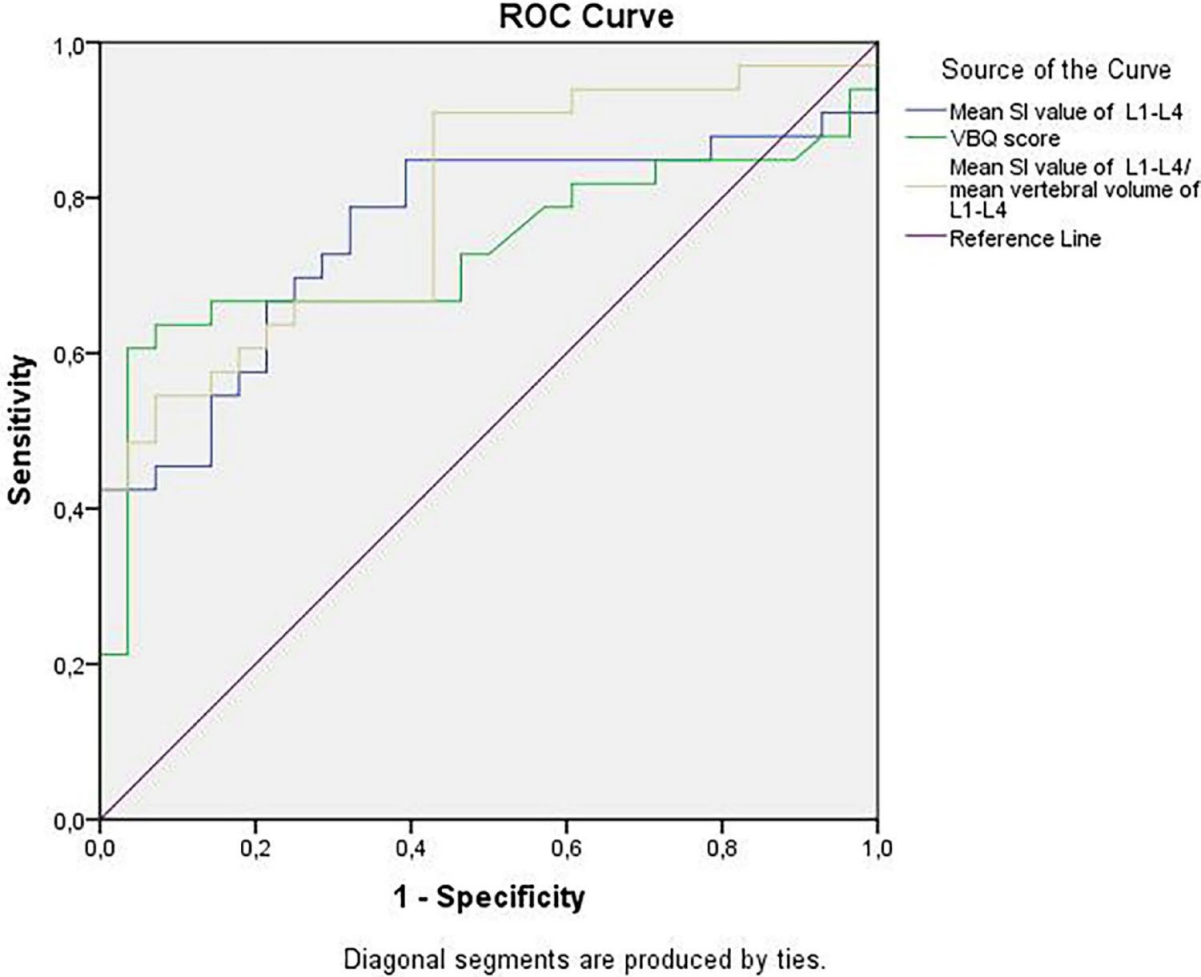
ROC curve analysis of the predictive values of MRI parameters for DXA-based osteoporosis diagnosis in childhood group (5–11 years). MRI signal intensity per unit volume of the vertebral body had the highest predictive power for low bone mineral density (AUC: 0.792, %95 CI: 0.678–0.906; *p* < 0.001), optimal cut-off value: 16.01

ROC analysis of the diagnostic performance of MRI parameters in the childhood group (Fig. 3) revealed that the MRI signal intensity per unit volume of the vertebral body had the highest predictive power for low bone mineral density (Table 2). Accordingly, an MRI signal intensity per unit volume of the vertebral body cut-off value of 16.01unit volume predicted low bone mineral density with 90.9% sensitivity and 57.1% specificity (AUC: 79.2%). On the other hand, a mean L1-L4 MRI signal intensity cut-off value of 258.3 predicted low bone mineral density with 75.8% sensitivity and 67.9% specificity (AUC: 75.8%) and a mean VBQ score cut-off value of 2.47 predicted low bone mineral density with 66.7% sensitivity and 64.3% specificity (AUC: 73.2%).

**Table 2:** The Results of the ROC Curve Analysis of the Predictive Values of the MRI-based Parameters for Osteoporosis Diagnosis in the Childhood Group

Test Variable	AUC	Std. Error	Cut-off Value	Sensitivity	Specificity	*p* value
MRI Signal Intensity Per Unit Volume of the Vertebral Body	0.792	0.057	16.01	0.909	0.571	**0.001**
Mean MRI Signal Intensity of the L1-L4 Vertebra Corpus	0.758	0.064	258.3	0.758	0.679	**0.001**
VBQ score	0.732	0.068	2.47	0.667	0.643	**0.002**

AUC: Area Under the Curve, *VBQ: Vertebral Bone Quality.* *p*-values in bold indicate statistical significance (*p* ≤ 0.05)

### Comparison of low bone mineral density patients and control subjects in the adolescence group

The adolescence (> 12 years) group consisted of 91 cases, 73 of whom were from the patient group, including 37 males and 36 females, and 18 from the control group, including 10 males and 8 females. The mean DXA Z scores of adolescence low bone mineral density patients and control subjects were − 3.44 and − 0.55, respectively.

In terms of MRI parameters, mean L1-L4 MRI signal intensity (238.5 vs. 195.9; *p* = 0.002) and MRI signal intensity per unit volume of the vertebral body (13.9 vs. 7.4; *p* < 0.001) were found to be significantly higher in the adolescence patient group than in the adolescence control group. On the other hand, there was no significant difference between these groups in VBQ score (2.3 vs. 2.2; *p* = 0.312).

**Fig. 4:**
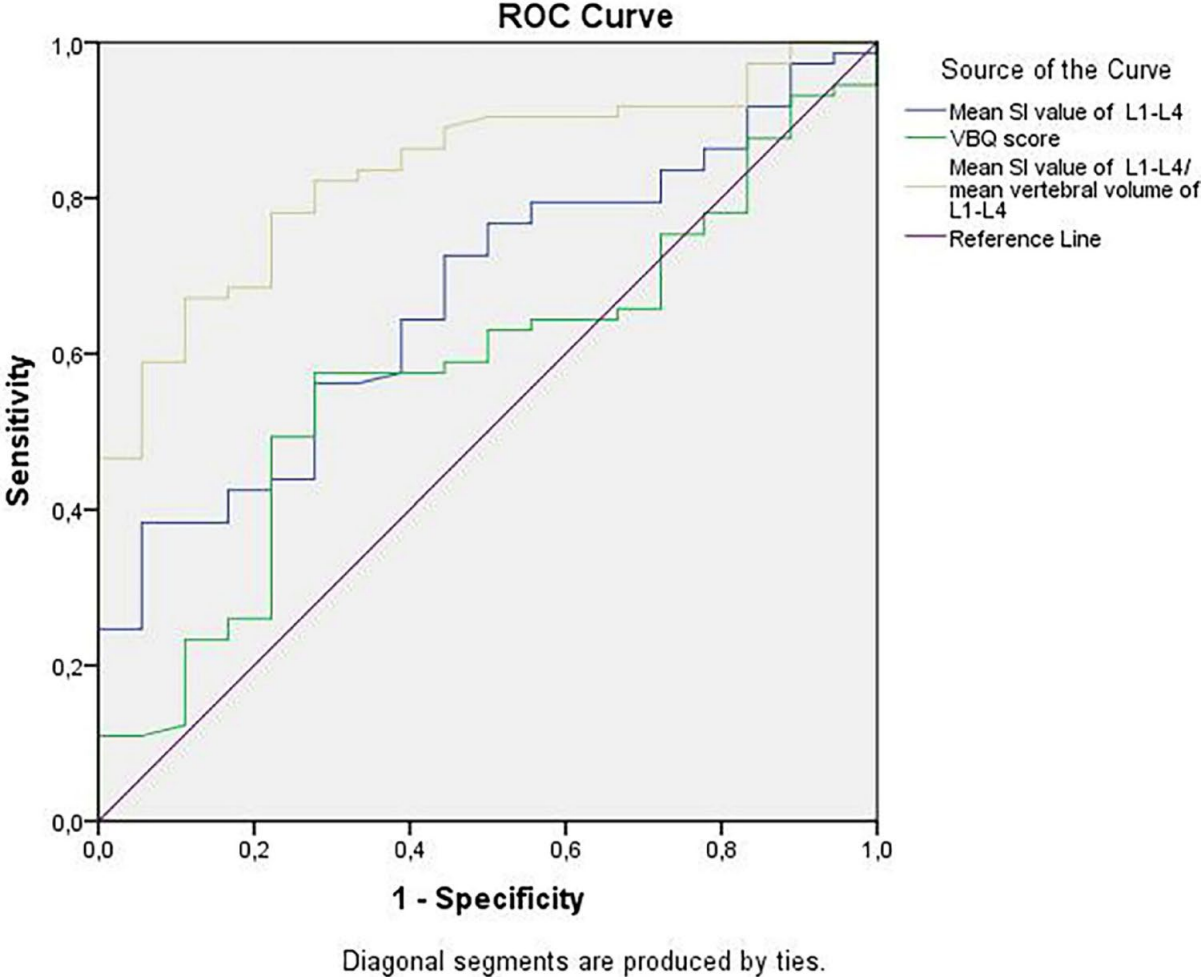
ROC curve analysis of the predictive values of MRI parameters for DXA-based osteoporosis diagnosis in adolescence group (> 12 years). MRI signal intensity per unit volume of the vertebral body had the highest predictive power for low bone mineral density (AUC: 0.836, 83.6% sensitivity and 66.7% specificity), optimal cut-off value:7.72

ROC analysis of the diagnostic performance of MRI parameters in the adolescence group (Fig. 4) revealed that the MRI signal intensity per unit volume of the vertebral body had the highest predictive power for low bone mineral density (Table 3). Accordingly, an MRI signal intensity per unit volume of the vertebral body cut-off value of 7.72 predicted low bone mineral density with 83.6% sensitivity and 66.7% specificity (AUC: 83.6%). On the other hand, a mean L1-L4 MRI signal intensity cut-off value of 179.5 predicted low bone mineral density with 72.6% sensitivity and 55.6% specificity (AUC: 67.2%).

**Table 3:** The Results of the ROC Curve Analysis of the Predictive Values of the MRI-based Parameters for Osteoporosis Diagnosis in the Adolescence Group

Test Variable	AUC	Std. Error	Cut-off Value	Sensitivity	Specificity	*p* value
MRI Signal Intensity Per Unit Volume of the Vertebral Body	0.836	0.045	7.72	0.836	0.667	**0.001**
Mean MRI Signal Intensity of the L1-L4 Vertebra Corpus	0.672	0.063	179.5	0.726	0.556	**0.024**
VBQ score	0.577	0.07	2.17	0.63	0.5	0.312

AUC: Area Under the Curve, *VBQ: Vertebral Bone Quality.* *p*-values in bold indicate statistical significance (*p* ≤ 0.05)

Vertebral volume is an important factor to consider, particularly in our Adolescent group where a wide range of sizes is expected. To rigorously evaluate this potential confounding effect, we performed an analysis of covariance (ANCOVA) to determine if vertebral volume independently affects our signal intensity/volume parameter and whether group differences persist after accounting for this variable.

The ANCOVA model was highly significant (R² = 0.86, F(4,86) = 135.3, *p* < 0.001). Vertebral body volume remained a strong covariate (*p* < 0.001). Age showed a moderate effect (*p* = 0.011), whereas sex was not significant (*p* = 0.59). Importantly, the adjusted mean SI/volume values were 3.84 (95% CI: 2.33–5.35) in the control group and 20.48 (95% CI: 19.80–21.17) in the Low BMD group.

These results demonstrate that while SI/volume is indeed influenced by vertebral body volume, the between-group differences persisted after adjusting for vertebral size and other covariates. Therefore, although part of the observed variability in the Adolescent group may reflect differences in vertebral volume, the adjusted analyses confirm that SI/volume retains its discriminatory value independent of vertebral size.

### Comparison of low bone mineral density patients and control subjects

**Fig. 5:**
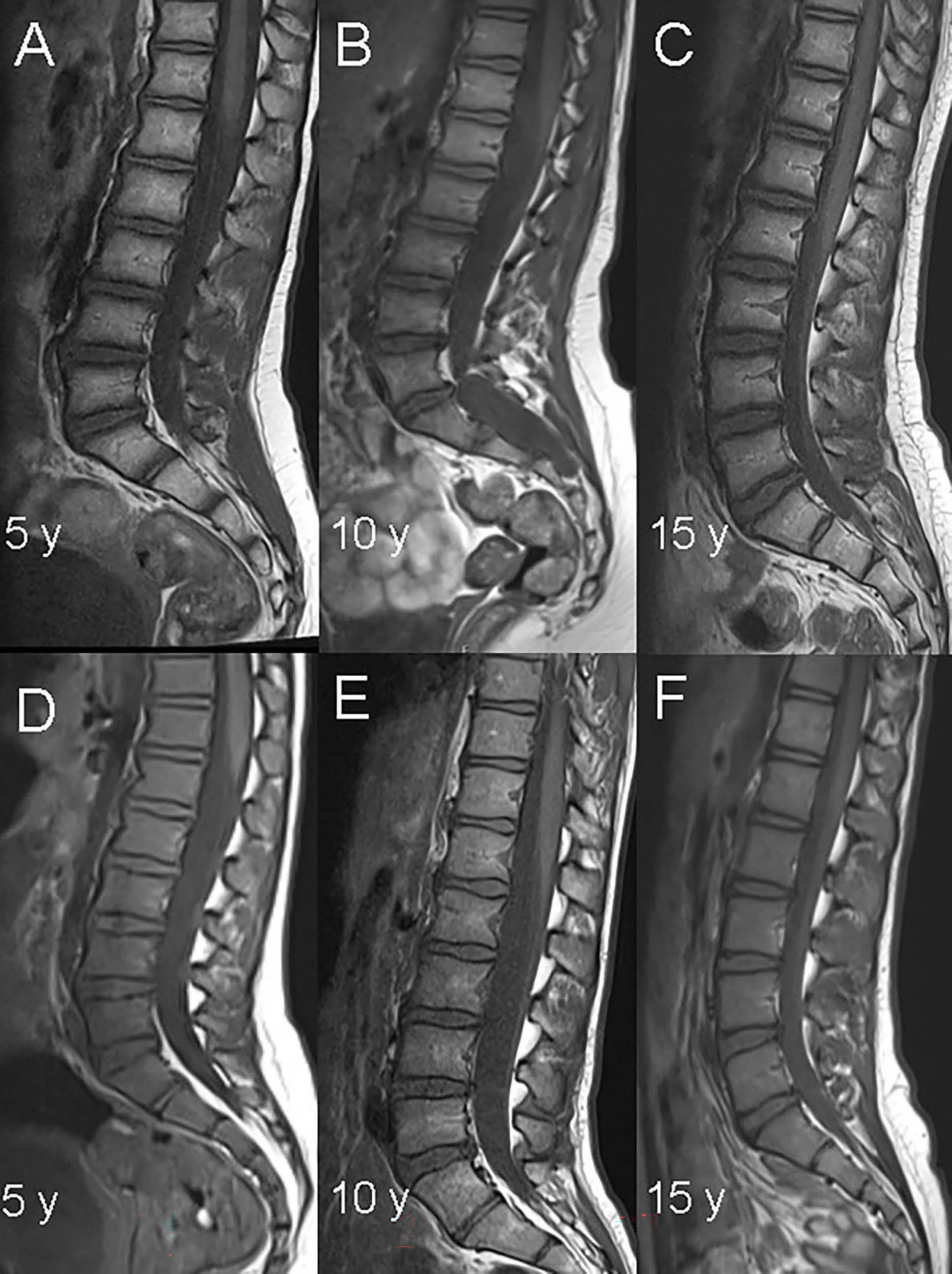
Representative sagittal T1-weighted MR images of patients with low bone mineral density at ages 5, 10, and 15 years (**A–C**, respectively) and age-matched controls (**D–F**, respectively)

Comparison of patient and control groups independent of age groups revealed that the mean L1-L4 MRI signal intensity (259.3 vs. 223.2; *p* = 0.011) and MRI signal intensity per unit volume of the vertebral body (19.27 vs. 13.87; *p* = 0.011) were significantly higher in the patient group than in the control group. On the other hand, there was no significant difference between the groups in terms of VBQ scores (2.47 vs. 2.27; *p* = 0.086). Representative sagittal T1-weighted MR images of patients with low BMD and age-matched controls are shown in Fig. 5.

Correlation analysis revealed a weak negative relationship between DXA Z score and mean L1-L4 MRI signal intensity (*r* = -0.221) and MRI signal intensity per unit volume of the vertebral body (*r* = -0.259).

ROC analysis revealed that the mean L1-L4 MRI signal intensity and the MRI signal intensity per unit volume of the vertebral body significantly predicted the presence of low bone mineral density in the pediatric population (*p* < 0.05 for both cases), whereas the VBQ score did not (*p* > 0.05).

### Subgroup analysis: non–marrow disorder cohort

In the subgroup analysis restricted to patients without marrow-affecting disorders, the results were highly consistent with the primary analysis. The mean L1–L4 MRI signal intensity remained significantly higher in the patient group compared with the control group (269.3 vs. 223.2; *p* = 0.003). Similarly, MRI signal intensity per unit vertebral volume was again significantly elevated in patients (22.99 vs. 13.87; *p* < 0.001), and VBQ scores were significantly higher in patients than in controls (2.55 vs. 2.27; *p* = 0.002).

ROC analysis in this subgroup confirmed that MRI signal intensity per unit vertebral volume retained the highest predictive power for low bone mineral density. A cut-off value of 16.1 predicted low bone mineral density with 61.0% sensitivity and 76.1% specificity (AUC: 72.8%). By comparison, a mean L1–L4 MRI signal intensity cut-off of 266.5 yielded 50.0% sensitivity and 82.6% specificity (AUC: 66.8%), while a mean VBQ score cut-off of 2.73 predicted low bone mineral density with 38.0% sensitivity and 91.3% specificity (AUC: 64.9%).

Interobserver reproducibility was assessed in a subset of 20 cases measured independently by two readers. The intraclass correlation coefficients demonstrated good to excellent reliability across all quantitative parameters: L1–L4 SI to vertebral volume ratio (ICC = 0.93, excellent), mean L1–L4 SI (ICC = 0.88, good), VBQ score (ICC = 0.86, good), and vertebral volume (ICC = 0.83, good). These results confirm the reproducibility of our MRI-based measurements.

## Discussion

Our study demonstrated that MRI signal intensity per unit vertebral volume, a newly proposed parameter, had the highest diagnostic performance in identifying low bone mineral density among pediatric patients. This parameter outperformed both the conventional L1-L4 mean MRI signal intensity and the widely used vertebral bone quality (VBQ) score, particularly in the childhood group. Notably, it showed strong discriminatory power in both childhood and adolescence groups, with area under the curve (AUC) values exceeding 0.79. These findings suggest that MRI-based assessment of signal intensity adjusted for vertebral volume may provide a clinically valuable, radiation-free alternative to DXA in pediatric populations.

In recent years, magnetic resonance imaging (MRI)–based techniques have emerged as promising alternatives for evaluating bone quality in children, primarily due to their lack of ionizing radiation. In particular, chemical shift encoding–based MRI (CSE-MRI) enables the quantitative assessment of bone marrow fat through the proton density fat fraction (PDFF), a reproducible and validated parameter that has shown utility in diagnosing osteoporosis. Ruschke et al. demonstrated the reliability of six-echo CSE-MRI in quantifying vertebral bone marrow fat, reporting mean PDFF values of 17.6% ± 7.9 in healthy adults with excellent reproducibility (ICC > 0.95) [14]. Furthermore, Gassert et al. established age-specific reference values for vertebral PDFF in a pediatric cohort (*n* = 190, ages 5–18), finding mean PDFF values of 20.3% ± 5.1 which significantly increased with age (*p* < 0.001) [15]. Additional studies have linked increased bone marrow fat to decreased bone mineral density (BMD) in adolescents and children with chronic diseases, suggesting PDFF as a potential biomarker for bone quality deterioration in pediatric osteoporosis [16, 17]. Li et al. further confirmed PDFF’s diagnostic value in osteoporosis, showing significantly elevated PDFF values in osteoporotic patients compared to controls (35.2% ± 9.4 vs. 21.5% ± 6.1, *p* < 0.001) [18].

Despite the advantages of CSE-MRI and PDFF-based measurements, there remains a need for practical and accessible parameters that can be derived from routine MRI sequences without the requirement for specialized imaging protocols or post-processing tools. In this study, we propose the use of signal intensity per vertebral volume, calculated from standard T1-weighted lumbar spine images. This approach aims to provide a size-normalized signal measure that may reflect bone mineral content in the pediatric population. Future studies are needed to validate this parameter against advanced fat quantification techniques such as proton density fat fraction (PDFF) based on chemical shift-encoded MRI and to establish age-specific reference values for broader clinical application.

In recent studies, MRI-based quantitative parameters and various scoring systems derived from these parameters have come to the fore in predicting bone mineral density. In one of these studies evaluating the diagnostic performance of various modalities, Wei et al. reported that the diagnostic accuracy of the MRI-based VBQ score was higher than that of CT and DXA examinations and recommended using these methods in combination [19]. In another study, where the VBQ score was compared with the DXA T score, Ehresman et al. demonstrated that the VBQ score can be used in the diagnosis of osteoporosis with high accuracy [20]. In a meta-analysis published in 2024 that included 2981 patients and 23 studies, Yang et al. reported the sensitivity and specificity of the VBQ score in diagnosing osteoporosis as 77% and 65%, respectively, demonstrating that the VBQ score is an effective parameter in distinguishing between normal bone mineral density and bone mineral loss [21]. Huang et al. reported that the VBQ score had a moderate-to-strong correlation with the DXA T score and a moderate correlation with the CT Hounsfield unit (HU) value and that the VBQ score obtained from the S1 vertebra predicted low mineral density with 77% sensitivity and 70% specificity [22]. In their study comparing MRI-based VBQ score, Osteoporosis Self-Assessment Tool for Asians (OSTA), and CT-based HU tests in the diagnosis of preoperative osteoporosis in cases undergoing spinal surgery, Wang et al. reported that the diagnostic sensitivity and specificity of the VBQ score were higher than other diagnostic tests [23]. In comparison, we found that the VBQ score predicted low bone mineral density with 66.7% sensitivity and 64.3% specificity in the childhood group (5–11 years of age), consistent with the literature.

Ramos et al. reported that the MRI-based VBQ score in adolescent idiopathic scoliosis (AIS) cases with osteopenia and osteoporosis was significantly higher than that of the control group [2.5 standard deviation (SD) 0.4 vs. 2.1 SD 0.3] [7]. In another study conducted with the AIS patient group, Yang et al. revealed that the VBQ score of patients with low mineral density was significantly higher than that of the control group [8]. In contrast, we found no significant difference in VBQ score between osteoporosis patients and control subjects in the adolescence group (2.3 vs. 2.2; *p* = 0.312).

The fact that bone mineral density (g/cm²) and DXA score are affected by body size causes the bone mineral density of short children to be underestimated. Therefore, values adjusted for body or bone size, i.e., volumetric bone mineral density (vBMD, g/cm³) and HAZ values, should be used [3]. In this context, we proposed MRI signal intensity per unit volume of the vertebral body as a novel parameter to predict cases of low bone mineral density with a view to reducing the effect of body and vertebra size differences between individuals on bone signal intensity. This new MRI-based parameter we proposed outperformed the VBQ score in detecting low bone mineral density in the pediatric population in both childhood and adolescence groups.

Age-related alterations in bone marrow composition, together with the heterogeneous distribution of residual red marrow, may substantially influence local signal intensity and introduce sampling errors when the selected measurement area does not adequately represent the entire marrow compartment [24]. Moreover, interindividual differences in vertebral size can contribute to variability in the sampling process, further affecting measurement reliability. Accordingly, our rationale for normalizing signal intensity to vertebral volume was not to replicate the methodological principles of DXA, but rather to minimize geometry- and biology-related sources of bias and thereby enhance comparability across patients.

In a recent study, Ehresman et al. demonstrated that the MRI-based VBQ score has a higher prognostic value in predicting the risk of osteoporotic fractures compared to DXA-based bone mineral density [22–25]. Yin et al. also reported that the VBQ score was an independent risk factor for osteoporotic fracture and negatively correlated with the DXA T score [23–26]. In comparison, the correlation analysis, in which we included all age groups and bone mineral density groups, revealed that the MRI signal intensity per unit volume of the vertebral body was negatively correlated with the DXA Z score. On the other hand, we did not find any significant differences in DXA parameters, MRI-based quantitative parameters, and laboratory parameters between patients with and without a history of fracture. The limited significance of VBQ in adolescents may be related to imbalances in sample sizes between adolescent groups. Furthermore, the significantly larger vertebral volumes observed in the control group compared to osteoporotic patients may have limited the predictive power of imaging parameters. Additionally, age-related differences in bone marrow composition during adolescence may have reduced VBQ performance. In this age group, the persistence and heterogeneous distribution of residual red bone marrow may reduce the sensitivity of MR-based signal measurements [24].

Our study, which investigated the relationship between DXA-based Z score and MRI-based VBQ score, revealed that MRI signal intensity per unit volume of the vertebral body performs better than the VBQ score in determining low mineral density. In the pediatric population, bone marrow signal intensity undergoes age-related changes due to the gradual conversion from red to yellow marrow, and in some cases, heterogeneous residual red marrow may be observed. This heterogeneity can lead to variability in vertebral body signal intensity measurements depending on the method applied. Consequently, there remains uncertainty as to whether differences in signal intensity per vertebral volume and the diagnostic performance of the VBQ score are influenced by ROI placement. For this reason, in our study, considering the smallest vertebral body volume in the cohort, we determined the fixed ROI diameter to be 10 mm, which could be used to remain within the vertebral medulla in all cases. Care was taken to ensure that the center of the ROI was placed precisely at the midpoint of the vertebral body in the midsagittal plane for all cases. This approach was intended to reduce potential interobserver variability and to standardize manual measurements. However, this method has certain limitations, as fixed-diameter ROIs may encompass different proportions of the vertebra depending on its size, potentially introducing measurement bias.

In the pediatric group, the high sensitivity of MR signal intensity per unit volume of the vertebral body reduces the likelihood of missing cases with low BMD and may provide an advantage in terms of follow-up in patients with chronic disease requiring long-term monitoring. However, its relatively low specificity may increase false-positive rate, necessitating additional confirmatory tests. Therefore, using this parameter as a screening tool in the pediatric subgroup and confirming positive results with DXA may be an appropriate strategy.

A notable strength of the present study is its heterogeneous patient cohort, encompassing children with different predispositions to primary and secondary osteoporosis who required radiological evaluation. Interpretation of vertebral bone marrow signal intensity in the pediatric population requires a more nuanced approach compared with adults. In adults, bone marrow composition is relatively stable, and a gradual fatty conversion of the hematopoietic marrow with aging is expected. Consequently, hyperintensity on T1-weighted images is often equated with fatty marrow replacement. However, in children, bone marrow undergoes continuous and dynamic processes of conversion and reconversion in response to both physiological and pathological demands. Therefore, attributing vertebral T1 hyperintensity solely to fatty marrow, as commonly done in adult studies, may not accurately reflect the pediatric context [27].

Several clinical conditions can alter this physiologic background. In chronic anemias such as thalassemia, sustained hematopoietic demand leads to reconversion and marrow hyperplasia, typically presenting as decreased T1 signal [28]. Conversely, after chemotherapy and/or radiotherapy, fatty marrow replacement is frequently observed and manifests as T1 hyperintensity [29]. Hematopoietic reactivation—triggered by anemia, smoking, or systemic stress—can also lower T1 signal intensity [30]. Future research should focus on age- and disease-stratified analyses and incorporate quantitative imaging biomarkers such as proton density fat fraction (PDFF) to validate qualitative MRI interpretations [31]. Such approaches will provide more reliable and clinically meaningful assessments of pediatric bone marrow alterations. In this context, we believe that our study will pioneer in determining MRI-based osteoporosis parameters to be used in the diagnosis of osteoporosis, regardless of the disease characteristics and facilitating factors of individuals, and will contribute to reducing the use of methods that involve ionizing radiation.

Besides the strengths of this study, it has several limitations that should be considered when interpreting its results. First, the study’s single-center and retrospective design created a potential selection bias that may affect the generalizability of its findings to broader pediatric populations. Although the study’s retrospective design limited our ability to standardize imaging protocols and data collection processes, we sought to minimize this adverse effect using consistent measurement techniques. Secondly, we could not include more subjects in our control group, particularly in the adolescence group, due to ethical concerns regarding subjecting asymptomatic healthy children to DXA measurements. This imbalance between the number of osteoporotic patients (*n* = 73) and control subjects (*n* = 18) in the adolescence group may have affected the statistical power of comparisons in this age group. Thirdly, although we evaluated a number of MRI parameters in our study, we did not use advanced MRI techniques such as quantitative MR spectroscopy or diffusion-weighted imaging, which may have provided additional information about bone microstructure and marrow composition. Another important limitation of our study is the reliance on T1-weighted signal without quantitative fat-fraction validation (e.g., PDFF or MR spectroscopy), which limits our ability to distinguish physiologic fatty conversion from red-marrow hyperplasia. Given the dynamic nature of pediatric bone marrow and its potential to reconvert under conditions such as anemia or systemic stress, some observed T1 signal changes may reflect hematopoietic hyperplasia rather than fatty replacement. Future studies should integrate quantitative imaging biomarkers with hematologic parameters and stratify analyses by age, disease status, and treatment history to establish more precise normative references and improve the reliability of pediatric bone marrow assessment. And lastly, a potential limitation of our approach is the use of fixed ROI sizes (10 mm for vertebrae and 5 mm for CSF), which may not fully account for age-related differences in vertebral dimensions. Although we placed ROIs centrally and averaged measurements across multiple vertebrae to minimise sampling variability, this method may still introduce an age-dependent bias. Scaling ROI size to vertebral dimensions could provide a more standardised and potentially more accurate strategy for future studies.

These limitations should be addressed in future prospective, multicenter studies with more extensive, balanced cohorts. Longitudinal studies assessing the predictive values of MRI-based parameters for fracture risk and their use in monitoring treatment response would contribute to incorporating these techniques into clinical practice. Additionally, establishing standardized imaging parameters and age-specific reference values for MRI-based bone quality assessments will further expand its use in clinical practice.

## Conclusions

In conclusion, our study revealed that MRI signal intensity per unit volume of the vertebral body, one of the MRI-based parameters, effectively determines low bone mineral density in the pediatric population. This novel MRI-based parameter predicted low bone mineral density, demonstrating better diagnostic performance than the VBQ score in both childhood and adolescence age groups in a heterogeneous pediatric cohort that included various primary disease groups. In addition, correlation analysis revealed that it was negatively correlated with the DXA Z score (*r* = -0.259, *p* < 0.05), which quantitatively supported its diagnostic value.

Contrary to the previous studies in the literature that focused on homogeneous adult populations with specific pathologies, the fact that our study included a heterogeneous pediatric cohort with a variety of diseases predisposing to osteoporosis increased the generalizability of its findings.

The MRI signal intensity per unit volume of the vertebral body may have potential utility as a biomarker for radiation-free monitoring in paediatric patients at risk of osteoporosis. These findings have significant clinical implications in that they position MRI as a radiation-free alternative to DXA and CT for bone mineral density assessment in pediatric populations, particularly in those that require long-term follow-up and are especially sensitive to the effects of radiation exposure. Furthermore, MRI-based assessment allows for the simultaneous evaluation of spinal morphology alongside quantitative assessment of bone quality. However, prospective longitudinal studies are needed to validate its predictive value for incident osteoporotic fractures and to determine its utility for monitoring treatment response.

## Data Availability

The datasets generated and analyzed during the current study are not publicly available due to institutional data protection regulations but are available from the corresponding author upon reasonable request, subject to approval from the institutional review board.
